# Non-Smc element 5 (Nse5) of the Smc5/6 complex interacts with SUMO pathway components

**DOI:** 10.1242/bio.018440

**Published:** 2016-05-23

**Authors:** Denise E. Bustard, Lindsay G. Ball, Jennifer A. Cobb

**Affiliations:** Department of Biochemistry and Molecular Biology, Robson DNA Science Centre, Arnie Charbonneau Cancer Institute, Cumming School of Medicine, University of Calgary, 3330 Hospital Drive N.W., Calgary, Alberta, Canada T2N 4N1

**Keywords:** Smc5/6 complex, SUMO, Ubc9, PIAS E3 SUMO ligases

## Abstract

The Smc5/6 complex in *Saccharomyces cerevisiae* contains six essential non-Smc elements, Nse1-6. With the exception of Nse2 (also known as Mms21), which is an E3 small ubiquitin-like modifier (SUMO) ligase, very little is understood about the role of these components or their contribution to Smc5/6 functionality. Our characterization of Nse5 establishes a previously unidentified relationship between the Smc5/6 complex and factors of the SUMO pathway. Nse5 physically associates with the E2 conjugating enzyme, Ubc9, where contacts are stabilized by non-covalent interactions with SUMO. SUMO also mediates the interactions between Nse5 and the two PIAS family E3 SUMO ligases, Siz1 and Siz2. Cells carrying the *nse5*-ts1 allele or lacking either *SIZ1* or *SIZ2* exhibit a reduction in Smc5 sumoylation upon MMS treatment and demonstrate functional redundancy for SUMO mediated events in the presence of DNA damage. Overall, given the extensive connection between Nse5 and components of the SUMO pathway, we speculate that one function of the Smc5/6 complex might be as a scaffold center to enable sumoylation events in budding yeast.

## Abstract (precis)

**Summary:** Here we characterize Nse5, a component of the Smc5/6 complex. Nse5 interacts with the PIAS E3 SUMO ligases, Siz1 and Siz2, and regulates Smc5 sumoylation.

## INTRODUCTION

The Smc5/6 complex is a member of the structural maintenance of chromosomes (SMC) family of proteins that includes cohesin and condensin. These complexes function in part by providing organization to chromatin during multiple processes including transcription, chromosome condensation and segregation, and DNA repair (Jeppsson et al., 2014; Tapia-Alveal et al., 2014). The Smc5/6 complex includes two SMC components, Smc5 and Smc6, and six non-Smc elements, Nse1-6, all of which are essential for survival in the budding yeast *Saccharomyces cerevisiae* (Zhao and Blobel, 2005; Ben-Aroya et al., 2008; Duan et al., 2009b).

The binding location of Nse1-4 within the complex is conserved between organisms; however, the position of Nse5-Nse6, which forms a heterodimeric subcomplex, is more divergent. In *Schizosaccharomyces pombe* (fission yeast), Nse5 and Nse6 were mapped to bind the head region of Smc5 and Smc6 (Palecek et al., 2006), but in *Saccharomyces cerevisiae* (budding yeast), Nse5 and Nse6 were found to bind the hinge region of Smc5 and Smc6 (Duan et al., 2009b). High throughput yeast two hybrid (Y2H) studies in budding yeast identified a number of potential Nse5 binding partners including some components of the sumoylation machinery and the small ubiquitin modifier (SUMO) protein itself (Hazbun et al., 2003). In addition, we determined that even though Nse5 interacted with SUMO, it was not a target of sumoylation (Bustard et al., 2012). Here we generate additional mutant alleles of *NSE5* with the aim of understanding the physiological significance of Nse5-SUMO interactions and determining a role for Nse5 within the Smc5/6 complex.

Nse2 (hereafter referred to as Mms21) is a component of the complex that binds the coiled-coil domain of Smc5 (Duan et al., 2009a). Mms21 is an E3 SUMO ligase with a diverse range of targets including Smc5, Yku70, and Smc2 (Zhao and Blobel, 2005; Takahashi et al., 2008), and thus it potentially regulates a range of nuclear functions. Disruption of the Smc5 binding domain in Mms21, rather than it ligase domain, results in lethality. Thus, the essential function of Mms21 is likely its involvement in maintaining the conformation of the Smc5/6 complex, and not its SUMO ligase activity (Duan et al., 2009a).

SUMO family members have different names and the homolog in budding yeast is called *SMT3* (suppressor of mif two 3). Sumoylation is a posttranslational modification where SUMO is covalently attached to and detached from other proteins to modulate their functions. Prior to conjugation with target proteins, SUMO is first cleaved at its extreme C-terminus by Ulp1 to reveal a di-glycine motif (Johnson et al., 1997). Next, the E1-activating enzyme Aos1/Uba2 uses energy from ATP to form a SUMO-adenylate conjugate (Johnson et al., 1997). This SUMO-adenylate bond is necessary to form the thioester bond between SUMO and the E2 conjugating enzyme, Ubc9, which itself is able to conjugate SUMO to target proteins (Johnson and Blobel, 1997). Even though Ubc9 catalyzes sumoylation on its own, the process is greatly enhanced by the presence of an E3 SUMO ligase (Gareau and Lima, 2010). In budding yeast, there are four E3 SUMO ligases: PIAS family homologs, Siz1 and Siz2, which appear to catalyze the majority of sumoylation (Johnson and Gupta, 2001), Cst9 is a meiosis-specific ligase (Cheng et al., 2006), and Mms21, which as mentioned above, is a component of the Smc5/6 complex (Zhao and Blobel, 2005). Each of these ligases contain a Sp-RING domain that is essential for functionality, however the term ‘ligase’ is somewhat misinforming, as these E3 ligases do not actually perform an enzymatic reaction. Rather, it has been proposed that the role of the E3 is to orient the E2-thioester-SUMO complex in a conformation that favors the transfer of SUMO to the target protein (Geiss-Friedlander and Melchior, 2007). A SUMO acceptor site in targets has been mapped to be a lysine residue in the consensus ΨKxE where Ψ is an aliphatic residue (Mahajan et al., 1998; Matunis et al., 1998). Crystal structures revealed that the acceptor lysine sits in the catalytic site of Ubc9 and that the flanking residues interact along the surface of Ubc9 (Bernier-Villamor et al., 2002).

Our aim is to determine if Nse5 integrity is important during DNA damage, what its role is within the Smc5/6 complex, and its interactions with components of the SUMO pathway. We demonstrate genetic and physical interactions between Nse5 and SUMO pathway components and show that interactions between Nse5 and PIAS E3 ligases, Siz1 and Siz2, and with the E2 conjugating enzyme, Ubc9, are partially mediated by SUMO. Two temperature sensitive (ts) alleles, *nse5*-ts1 or *nse5*-ts2, show a marked decrease in Smc5 sumoylation and neither protein interacts with Smt3 in Y2H analysis. However unlike *nse5*-ts1, *nse5*-ts2 mutant cells did not show sensitivity to MMS, which uncouples a functional role for Smc5 sumoylation in response to MMS-induced DNA damage.

## RESULTS

### Genetic interactions and Smc5 sumoylation in *nse5*-ts1 mutants during MMS treatment

**Fig. 1.**
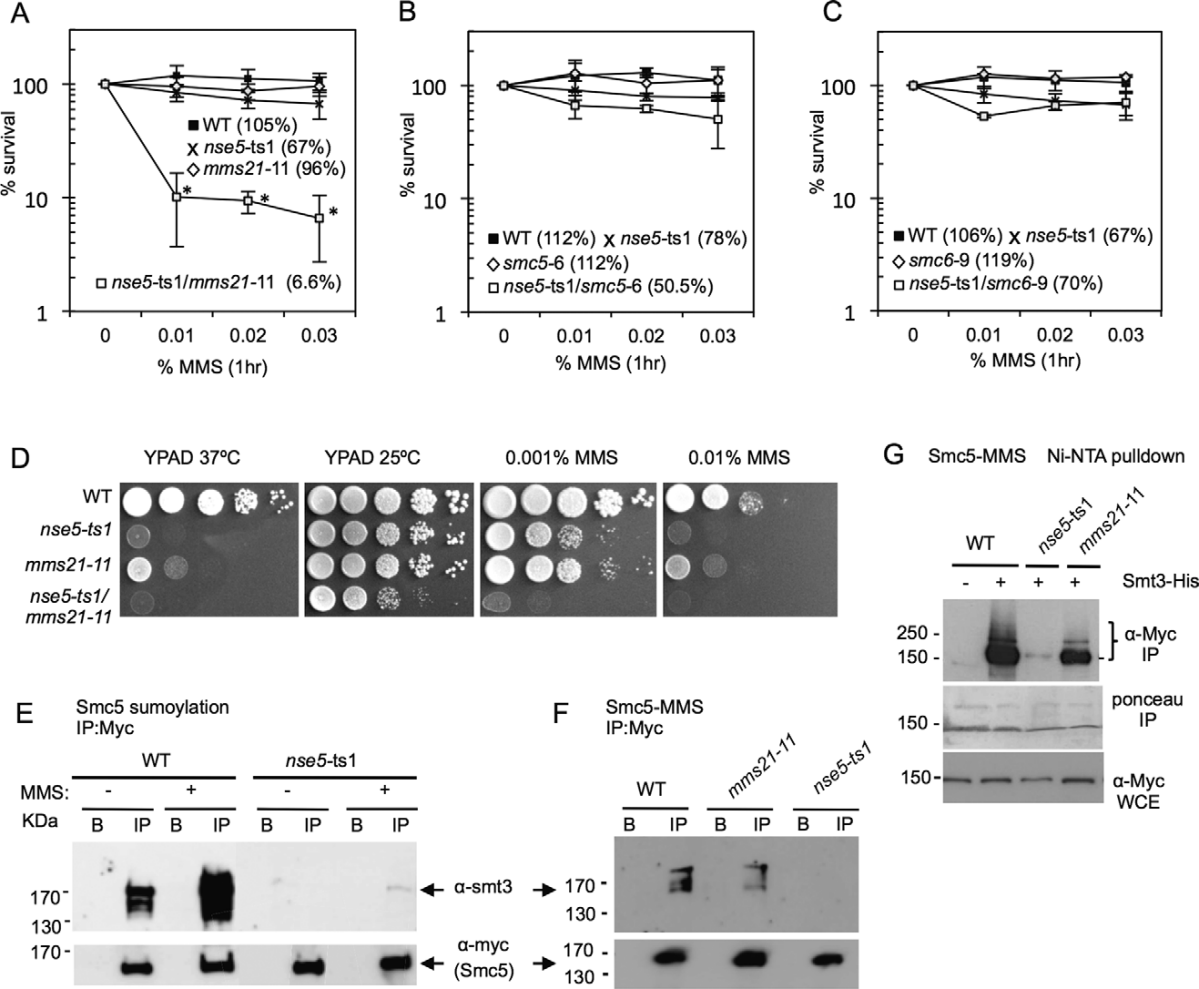
**Genetic interactions and Smc5 sumoylation with the *nse5*-ts1 mutant allele of the Smc5/6 complex.** (A-C) Cell viability was monitored as colony outgrowth from asynchronous cultures after transient exposure to MMS at indicated concentrations for 1 h at 30°C, with values normalized to survival at time point 0. The % survival reported in graph is value after treatment with 0.03% MMS. Data points represent the mean±s.d. at each concentration of MMS from *n*=3 experiments performed in technical duplicate. *P<0.01 compared to wild-type cells with values from a two-tailed *t*-test**.** Drop assays on YPAD at 37°C or 25°C without or with the indicted amounts of MMS were performed on 1:10 serial dilutions of exponentially growing cultures. The following strains were used: wild-type (WT) (JC471), *nse5*-ts1 (JC1321), *smc5*-6 (JC1844), *nse5*-ts1/*smc5*-6 (JC1843), *smc6*-9 (JC1358) and *nse5*-ts1/*smc6*-9 (JC1359), *mms21*-11 (JC1981) and *nse5*-ts1/*mms21*-11 (JC1319). (D) Drop assays (1:10 serial dilutions) from exponentially growing cultures were performed on YPAD medium at 37°C or 25°C without or with MMS at the indicated concentration for wild-type (JC1157), *nse5*-ts1 (JC1321), *mms21*-11 (JC1981) and *nse5*-ts1/*mms21*-11 (JC1319). (E,F) Smc5 sumoylation was analyzed in cells without or with 0.3% MMS at 25°C by immunoprecipitation of Myc-Smc5 followed by western blot analysis with anti-Smt3 at a concentration of 1:3000 (a kind gift from Zhao lab). Blots were then stripped and re-probed using anti-Myc antibody (9E10) at a concentration of 1:1000 to confirm equal loading. In a side-by-side comparison, Smc5 sumoylation increases upon MMS treatment and is reduced in *nse5*-ts1 cells (JC911) and *mms21*-11 (JC954) compared to wild-type (JC720). (G) As an alternative method to detect sumoylation, proteins were isolated by Ni-NTA affinity purification of His-Smt3 as described previously at 25°C (Wohlschlegel et al., 2004; Psakhye and Jentsch, 2012; Bustard et al., 2012) followed by western blotting with anti-Myc antibodies to visualize sumoylated proteins in cells containing Myc-tagged Smc5 with un-tagged Smt3 wild-type (JC720), or His8-tagged Smt3 in wild-type (JC1157), *nse5*-ts1 (JC1156) and *mms21*-11 (JC1155) after treatment with 0.3% MMS.

The Smc5/6 complex is involved in DNA replication and repair and characterizing the individual subcomponents of the complex will augment full understanding of how Smc5/6 works. To begin our characterization of the Nse5 component, we utilized the ts mutant, *nse5*-ts1, which is lethal at 37°C (Fig. S1A,B) (Bustard et al., 2012; Ben-Aroya et al., 2008). We combined this allele with other mutants in the Smc5/6 complex. Upon treatment with 0.03% MMS for 1 h, there was a loss in viability to ∼7% when *nse5*-ts1 was combined with *mms21*-11 compared to 67% and 96% for the *nse5*-ts1 and *mms21*-11 single mutants, respectively (Fig. 1A). This synergistic loss of viability was not observed when *nse5*-ts1 was combined with two other complex mutants, *smc5*-6 or *smc6*-9 (Fig. 1B,C), suggesting a potential for overlap in the functionality of Nse5 and Mms21. This sensitivity was also observed when cells were grown on plates containing a low concentration of 0.001% MMS (Fig. 1D). Other genetic interactions were also observed, for example *nse5*-ts1/*nse3*-1 double temperature-sensitive mutants showed moderate sensitivity over the single alleles (Fig. S1C) and cells harboring *nse5*-ts1/*nse4*-2 double mutants exhibited MMS sensitivity similar to levels observed with *nse5*-ts1/*mms21*-11 (Fig. S1C). In contrast to Mms21, however, a defined role for Nse4 within the complex is currently unclear and therefore was not further investigated in this study. As Mms21 is SUMO ligase (Zhao and Blobel, 2005) and Nse5 has potential ties to the SUMO pathway (Hazbun et al., 2003; Bustard et al., 2012), we wanted to investigate if Nse5 mediates the sumoylation of certain target proteins, such as Smc5, after MMS exposure.

We measured Smc5 sumoylation by western blot analysis with α-Smt3 (SUMO) on immuno-precipitated Smc5^Myc^±0.3% MMS treatment (Fig. 1E). Consistent with previous reports (Cremona et al., 2012; Bermudez-Lopez et al., 2015), sumoylation in wild-type cells, which is detected in unchallenged condition, markedly increases as after 1 h of exposure to MMS (Fig. 1E). Under conditions of MMS treatment, the level of Smc5 sumoylation was substantially reduced in *nse5*-ts1 mutants (Fig. 1E) and was even lower than what was observed in *mms21*-11 mutant cells (Fig. 1F, showing a lighter exposure for α-smt3 than in Fig. 1E to distinguish the levels sumoylation between WT and *mms21*-11). Due to limited antibodies, hereafter the status of sumoylation was detected using 8His-tagged Smt3 strains, where Smt3 is Ni-NTA purified under denaturing conditions. This method has been routinely used (Wohlschlegel et al., 2004; Psakhye and Jentsch, 2012; Ferreira et al., 2011; Bustard et al., 2012), and it was reassuring that the two approaches gave comparable results. The intensity of multiple bands migrating above 150 kDa, which represents the sumoylated form of Smc5, were reduced in the two mutants (Fig. 1G), with the reduction in Smc5 sumoylation in *nse5*-ts1 mutant cells appearing more pronounced than that observed with cells harboring the *mms21*-11 allele (Fig. 1G).

### Nse5 interacts with other components of the sumoylation pathway

**Fig. 2.**
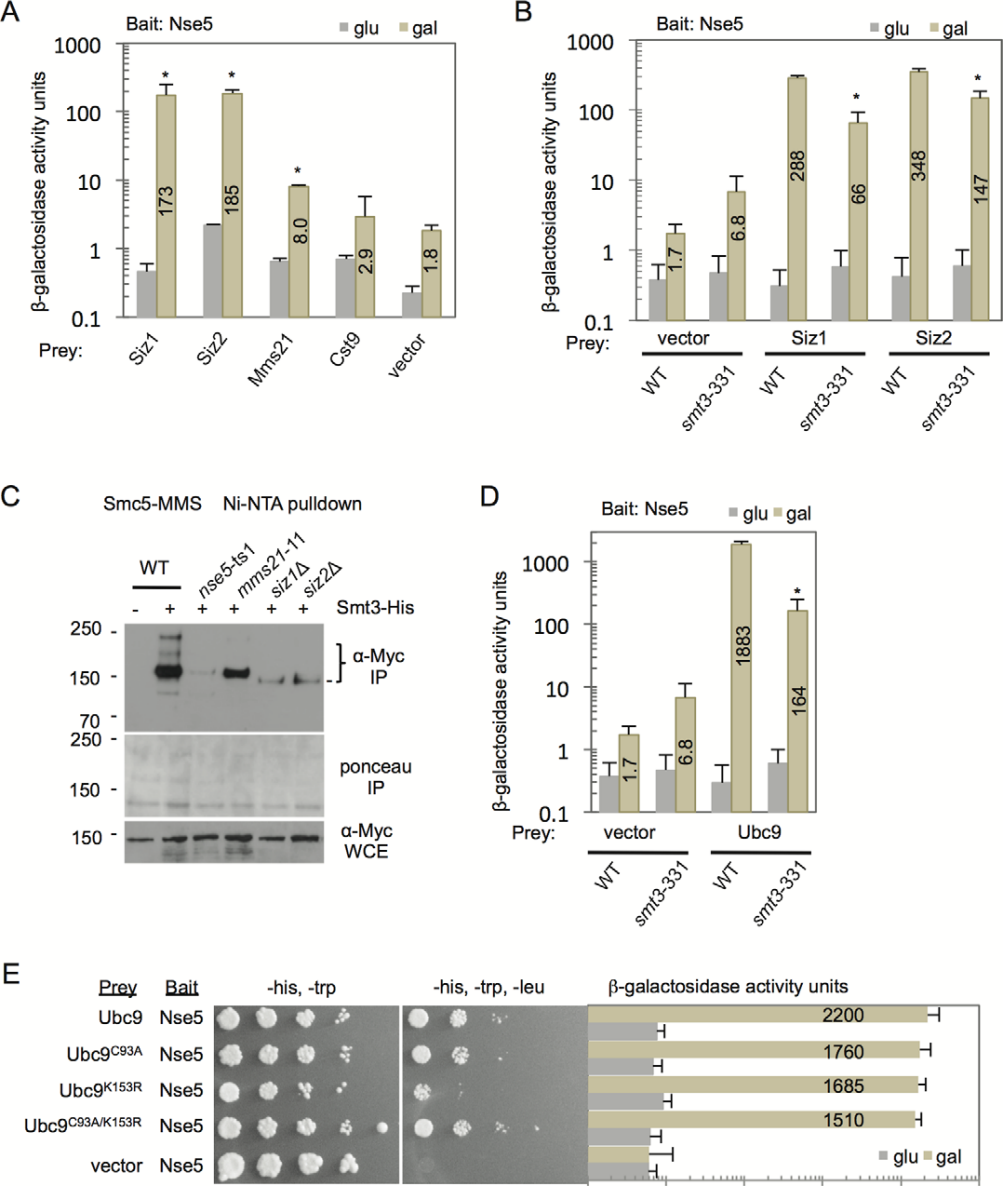
**Nse5 interactions with the PIAS family of E3 SUMO ligases, Siz1 and Siz2, and the E2-conjugating enzyme, Ubc9, is mediated by SUMO.** (A) Two-hybrid analysis to determine interactions between bait: Nse5-LexA (J-038) vector and prey vectors with the four budding yeast SUMO ligases: either empty vector (J-1493), Siz1- (J-096), Siz2- (J-047), Mms21- (J-050) and Cst9-AD (J-095). Error bar represent the standard deviation from the mean of *n*=3 biological experiments performed in technical triplicate. *P<0.01; statistically different interaction between Nse5 and the E3 ligases than with the interaction between Nse5 and the vector alone (background), from a two-tailed *t*-test in A. (B) Two-hybrid analysis as described in A comparing wild-type (JC470) and *smt3*-331 (JC2758) mutant cells. *P<0.01; statistically different interaction between Nse5 with Siz1 or Siz2 in cells harboring the *smt3*-11 mutation compared to the same interactions measured in wild-type cells, from a two-tailed *t*-test. Representative western blots in Fig. S2A show the expression from the two-hybrid constructs. (C) Using the His-Smt3 Ni-NTA purification system, sumoylated Smc5 was purified from cells endogenously Myc-tagged Smc5 with un-tagged Smt3 wild-type (JC720), or His8-tagged Smt3 wild-type (JC1157), *nse5*-ts1 (JC1156), *mms21*-11 (JC1155), *siz1Δ* (JC2527), and *siz2Δ* (JC1964) mutant cells following 0.3% MMS treatment at 25°C. (D) Two-hybrid analysis was performed to determine interactions between bait: Nse5-LexA (J-038) and prey vectors: either empty pJG4-6 vector (J-1493) or Ubc9-AD (J-042) in WT (JC470) or *smt3*-331 (JC2758) cells. *P<0.01; statistically different interaction between Nse5 with Ubc9 in cells harboring the *smt3*-11 mutation compared to the same interactions measured in wild-type cells, from a two-tailed *t*-test. All interaction between Nse5 and Ubc9 were statistically different than the interaction between Nse5 and the vector alone (background) in both wild-type and *smt3*-11 cells with *P*<0.01. (E) Two-hybrid analysis showed no statistically significant differences in interactions between bait: Nse5-LexA (J-038) with the prey constructs: Ubc9 (J-042, wild-type), Ubc9-C93A (J-108, catalytic site mutant), Ubc9-K153R (J-101, SUMO-acceptor site mutant), Ubc9-C93A/K153R (J-110, SUMO-acceptor site and catalytic site double mutant). All interactions were significantly different than background levels with Nse5-LexA (J-038) and empty vector pJG4-6 (J-1493). Western blot analysis in Fig. S2B shows the expression from two-hybrid constructs at comparable levels.

Nse5 was previously shown to interact with SUMO (Hazbun et al., 2003; Bustard et al., 2012) and from the same high throughput Y2H screen (Hazbun et al., 2003), Nse5 was also reported to potentially interact with the E3 SUMO ligase, Siz2, and the E2 conjugating enzyme Ubc9. To verify these observations and expand these analyses of Nse5, Y2H was performed between Nse5 and the four known E3 SUMO ligases in budding yeast. Statistically significant interactions were observed between Nse5 with all E3 ligases except Cts9, the meiosis-specific E3 (Fig. 2A) (Cheng et al., 2006).

The level of binding between Nse5 and Mms21 was significantly higher than background, (i.e. between Nse5 with vector alone), however it was ∼5% of the levels measured with Siz1 and Siz2 (Fig. 2A). These data are consistent with Nse5 and Mms21 binding distinct sites of Smc5, where Nse5 interacts at the hinge and Mms21 binds the coiled-coil region (Duan et al., 2009a,b), and suggests that while Nse5 and Mms21 are in the same complex, a strong direct interaction between these subcomponents is not observed.

Given Nse5 interacts with SUMO, we addressed whether the Nse5-Siz1 or -Siz2 interactions depended on SUMO. To this end we incorporated into our Y2H reporter strain a randomly generated temperature sensitive mutant of *SMT3*, *smt3*-331, where general sumoylation is impaired (Biggins et al., 2001; Srikumar et al., 2013). The interactions between Nse5 with Siz1 and Siz2 were reduced in cells harboring *smt3*-311 by 77% and 57%, respectively (Fig. 2B), even when protein overexpression levels were similar (Fig. S2A). In *smt3*-331 cells, SUMO is not completely depleted as it is an essential protein, thus it is unclear if the residual interactions are dependent on SUMO or not, however these data clearly show there is a correlation between decreased SUMO levels and decreased interactions between Nse5 and both PIAS family E3 SUMO ligases, Siz1 and Siz2. Given that the integrity of Nse5 was important for the SUMO status of Smc5 (Fig. 1F,G), we wanted to determine if the two ligases that interacted with Nse5 contributed to Smc5 sumoylation. A pronounced reduction in Smc5 sumoylation was observed in cells lacking either *SIZ1* or *SIZ2* after MMS treatment, with only a faint lower-migrating band visible (Fig. 2C). A previous quantitative mass spectrometry (MS) approach determined that Smc5 was not a target for sumoylation by Siz1 or Siz2 under unchallenged conditions (Albuquerque et al., 2013). However upon MMS treatment, our results suggest that functional redundancy with the E3 ligases might arise in response to genotoxic stress, as Smc5 sumoylation is reduced in *siz1*Δ and *siz2*Δ mutant cells.

We next determined binding between Nse5 and the E2 conjugating enzyme, Ubc9. A very robust interaction between Nse5 and Ubc9 was observed that was ∼5-fold greater than the interactions observed with Siz1 and Siz2 (Fig. 2D,E). As the interactions between Siz1 and Siz2 with Nse5 were partially dependent on SUMO, we wanted to address if the Nse5-Ubc9 interaction also depended on SUMO. We observed that the Nse5-Ubc9 interaction in *smt3*-331 mutants was less than 10% of the level in *SMT3*^+^ cells (Fig. 2D; Fig. S2B), suggesting that SUMO is also critical for Ubc9 binding Nse5.

SUMO and Ubc9 interact in multiple ways, the most obvious is via a thioester bond at the catalytic site of Ubc9. Transfer of this thioester bond between Ubc9 to the target protein is the last step in sumoylation (Schwarz et al., 1998). In order to test if this thioester bond was important for the Nse5-Ubc9 (SUMO-mediated) interaction, the Ubc9 catalytic cysteine residue 93 was mutated to alanine (Ubc9-C93A), and two-hybrid analysis was performed. The interaction between Nse5 and Ubc9 was only mildly reduced (Fig. 2E). Another way in which Ubc9 and SUMO can interact is through the sumoylation of Ubc9, which has been found to promote SUMO chain formation on target proteins (Klug et al., 2013). In order to determine whether Nse5 was specifically interacting with sumoylated Ubc9, the SUMO-acceptor lysine in Ubc9 was mutated to an alanine (Ubc9-K153R). Again, the Nse5- Ubc-K153R interaction was only mildly reduced, and the double mutant Ubc9-C93A/K153R was comparable to the single amino acid substitutions (Fig. 2E). None of the Ubc9 mutants were determined to be statistically different than wild type for interacting with Nse5. Previous mutational analysis of Ubc9 also determined that it can extensively interact with SUMO through non-covalent interactions (Bencsath et al., 2002), therefore it is plausible that non-covalent interactions between SUMO and Ubc9 support Nse5-Ubc9 binding, as mutations in the two characterized covalent SUMO-binding sites of Ubc9 had little impact on the Nse5-Ubc9 interaction(s).

### Nse5 point mutants were generated to identify residues interacting with SUMO

**Fig. 3.**
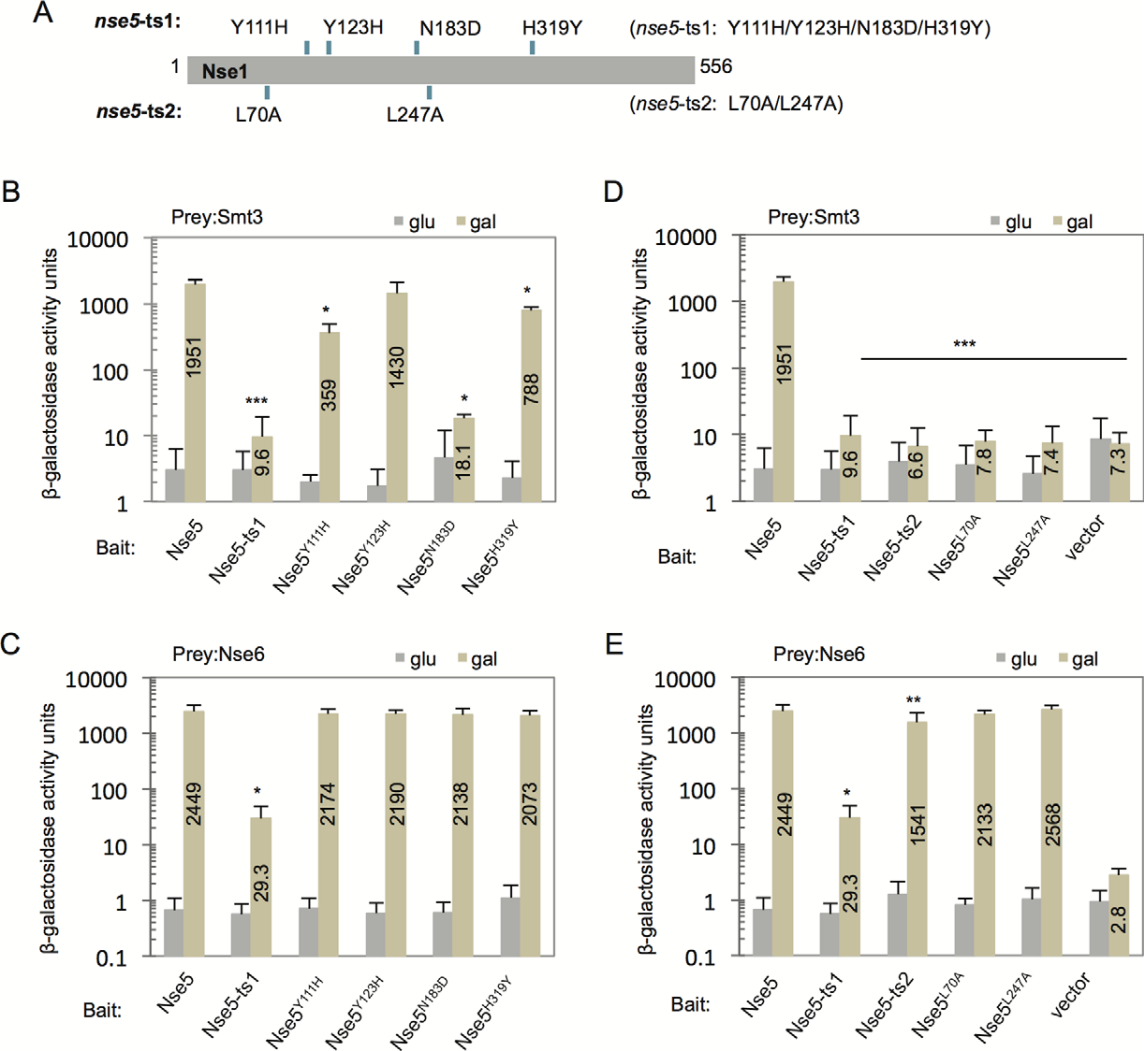
**Individual point mutants in *nse5*-ts1 and *nse5*-ts2 exhibit distinct changes in their binding to Smt3 and Nse6.** (A) Schematic comparing the four amino acid substitutions in Nse5-ts1: Y111H, Y123H, N183D, and H319Y and the two substitutions in Nse5-ts2: L70A and L247A. (B-E) Two-hybrid analysis to determine interactions between bait vectors: Nse5-LexA (J-038), Nse5-ts1- LexA (J-039), Nse5-Y111H-LexA (J-132), Nse5-Y123H-LexA (J-131), Nse5-N183D-LexA (J-121) Nse5-H319Y- LexA (J-120) and prey vectors: (B) Smt3-AD (J-043) and (C) Nse6-AD (J-063), or bait vectors: Nse5-LexA (J-038), Nse5-ts1- LexA (J-039), Nse5-ts2- LexA (J-067), Nse5-L70A-LexA (J-062), Nse5-L247A-LexA (J-066) and prey vectors: (D) Smt3-AD (J-043) and (E) Nse6-AD (J-063). Western blot analysis in Fig. S3A,B shows the expression from two-hybrid constructs at comparable levels. Error bar represent the standard deviation from the mean of *n*=3 biological experiments performed in technical triplicate. *P<0.05; **P<0.01; statistically different interaction between Nse5 mutants with Smt3 or Nse6 than with wild-type Nse5, from a two-tailed *t*-test. ***P<0.01; statistically different interaction between Nse5 mutants with Smt3 compared to wild-type Nse5, from a two-tailed *t*-test, and also not statistically different than Smt3 with the vector alone (not above background).

As Nse5 interacts with SUMO, and its interactions with Siz1, Siz2, and Ubc9 are mediated by SUMO, we wanted to identify critical residues in Nse5 that regulate its interactions with SUMO. The *nse5*-ts1 allele was generated by PCR-based random mutagenesis and contains 4 amino acid substitutions (Fig. 3A) (Bustard et al., 2012). Here we performed site-directed mutagenesis on the Y2H vector containing *NSE5* to generate the individual mutations collectively found in *nse5*-ts1 (Fig. 3A). Interactions between Smt3 (SUMO) with Nse5, wild-type and mutant proteins, were assessed after verification of expression levels (Fig. S3A). The Nse5-ts1 protein showed a marked reduction in its interaction with Smt3 to 0.5% of wild type (Fig. 3B) (Bustard et al., 2012). The individual point mutants showed varied interactions with Smt3. N183D and Y111H had the most severe defects, down to 1% and 18% of wild type respectively. There was also measurable, but less severe, reduction in the binding between Smt3 with H319Y to 40% of wild type (Fig. 3B), and downward trend, but a non-significant reduction, with Y123H (75%) compared to wild type (Fig. 3B). Next, we determined the ability of the point mutants to interact with Nse6, which is the binding partner of Nse5 within the complex. In contrast to Nse5-ts1, all mutants containing the individual amino acid substitutions interacted with Nse6 at levels comparable to wild type (Fig. 3C; Fig. S3B), indicating that the loss of Nse5-ts1 binding to Nse6 is not attributed to a single mutation. In a parallel set of experiments, we also characterized protein-protein interactions with *nse5*-ts2, another mutant that contains two substitutions, L70A and L247A (Fig. 3A) (Bustard et al., 2012). The point mutants and Nse5-ts2, which contains both L70A and L247A, showed a complete loss of interaction with Smt3 to the level of the vector control (Fig. 3D; Fig. S3A). In contrast, the individual alanine substitution, L70A and L247A, maintained a level of association with Nse6 that was indistinguishable from wild type (Fig. 3E; Fig. S3B) (Horigome et al., 2015). By comparison, the interaction of Nse5-ts2 with Nse6 was reduced to ∼63% of wild-type levels, which was more serve than the single mutants but was not as severe as Nse5-ts1 at only 1% of wild type (Fig. 3E). With Nse5 mutants that lose interaction with SUMO now identified, we next wanted to determine their impact *in vivo* on Smc5 sumoylation and cell survival upon MMS treatment.

### Mutants of *nse5* were integrated into the genome and assessed for Smc5 sumoylation and cell survival

**Fig. 4.**
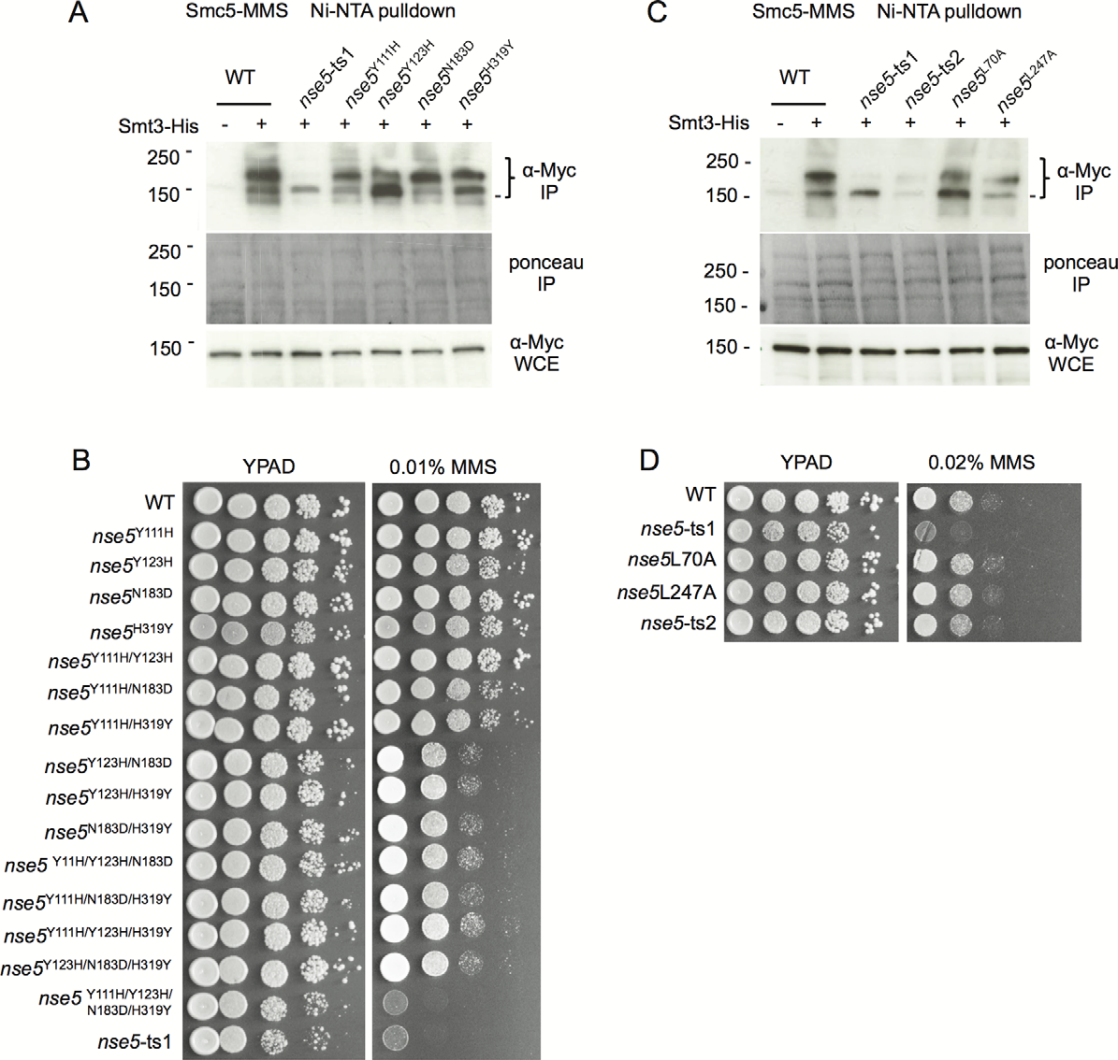
**Individual point mutants in *nse5*-ts1 and *nse5*-ts2 exhibit only minor changes in Smc5 sumoylation and MMS sensitivity.** (A) The His-Smt3 Ni-NTA purification system allowed for purification of sumoylated Smc5 following 0.3% MMS treatment at 25°C in cells containing endogenously Myc-tagged Smc5 with un-tagged Smt3 wild-type (JC720), or His8-tagged Smt3 in wild-type (JC1157), *nse5*-ts1 (JC1156), *nse5*-Y111H (JC3797), *nse5*-Y123H (JC3798), *nse5*-N183D (JC3799), or *nse5*-H319Y (JC-3800) mutants. (B) Drop assays (1:10 serial dilutions) with exponentially growing cultures were performed on YPAD±medium containing 0.01% MMS for wild-type (JC470), *nse5*^Y111H^ (JC2138), *nse5*^Y123H^ (JC2195), *nse5*^N183D^ (JC2139), *nse5*^H319Y^ (JC2140), *nse5*^Y111H/Y123H^ (JC2172), *nse5*^Y111H/N183D^ (JC2456), *nse5*^Y111H/H319Y^ (JC2196), *nse5*^Y123H/N183D^ (JC2405), *nse5*^Y123H/H319Y^ (JC2406), *nse5*^N183D/H319Y^ (JC2173), *nse5*^Y111H/Y123H/N183D^ (JC2192), *nse5*^Y111H/N183D/H319Y^ (JC2191), *nse5*^Y111H/Y123H/H319Y^ (JC2193), *nse5*^Y123H/N183D/H319Y^ (JC2407), *nse5*^Y111H/Y123H/N183D/H319Y^ (JC2194), and *nse5*-ts1 (JC1361) cells at 25°C. (C) Smc5 sumoylation was monitored as in A for un-tagged Smt3 wild-type (JC720), or His8-tagged Smt3 in wild-type (JC1157), *nse5*-ts1 (JC1156), *nse5*-ts2 (JC1884), *nse5*-L70A (JC2900), and *nse5*-L247A (JC3801) mutants. (D) Drop assays (1:10 serial dilutions) with exponentially growing cultures were performed on YPAD±medium containing 0.02% MMS for wild-type (JC1157), *nse5*-ts1 (JC1156), *nse5*-ts2 (JC1884), *nse5*-L70A (JC2900), and *nse5*-L247A (JC3801) cells at 25°C.

We generated strains where the *nse5* alleles, including the *NSE5* promoter, were integrated into the genome at the *URA3* locus and where endogenous *NSE5* was subsequently deleted so that only the mutant form was present. This was performed for all possible double and triple mutant substitutions in *nse5*-ts1. Only when all four substitutions were made, and the *nse5*-ts1 sequence was reconstructed, that substantial MMS sensitivity was observed (Fig. 4B). We also monitored Smc5^Myc^ sumoylation in strains containing 8His-tagged Smt3 by Ni-NTA purification. In contrast to *nse5*-ts1, which showed major defects in Smc5 sumoylation, only the nse5^Y111H^ and nse5^Y123H^ mutants displayed minor, but visible decreases in the slowest migrating band, indicating reduced sumoylation (Fig. 4A). However, the changes in Smc5 sumoylation showed no correlation with the changes in Smt3-Nse5 interactions. For example, Nse5-N183D showed reduced binding to Smt3, but in *nse5*^N183D^ cells, Smc5 sumoylation and MMS sensitivity looked indistinguishable from wild type (Fig. 3B, Fig. 4A,B). Taken together, these data suggest that multiple, or all substitutions in the protein expressed from the *nse5*-ts1 allele are required to reproduce the loss of Smc5 sumoylation and MMS sensitivity.

The alleles of *nse5*-ts2, nse5^L70A^, and nse5^L247A^ were also integrated into the genome and assessed for Smc5 sumoylation. Similar to *nse5*-ts1, *nse5*-ts2 mutant cells showed a marked loss in Smc5 sumoylation (Fig. 4C). Individually, the *nse5*^L70A^ and *nse5*^L247A^ alleles showed a decrease in sumoylation, however the reduction was not as severe as that measured in *nse5-ts2* mutant cells (Fig. 4C). These results demonstrate that nse5 mutants can be generated, which have reduced binding to Smt3 and compromised Smc5 sumoylation, but which still interact with Nse6 (and by extension the Smc5/6 complex). However, and surprising to us, cells harboring *nse5*-ts2 or the individual point mutations exhibited no sensitivity to MMS (Fig. 4D), indicating Smc5 sumoylation is dispensable for cell survival after MMS exposure. These results do not question the importance of the Smc5/6 complex in DNA repair or genome maintenance, but rather the role of Smc5 sumoylation in these processes. Until a clear function for Smc5 sumoylation is identified via mutagenesis of the acceptor lysine(s) on Smc5 we halted further analysis of Smc5 sumoylation in the various double and triple combinations of the *nse5-ts1* allele.

## DISCUSSION

Here we have partially characterized the Nse5 component of the Smc5/6 complex. Our work investigates the molecular requirements for Smc5 sumoylation; which, similar to multiple proteins involved in homologous recombination, is strongly induced by DNA damage (Fig. 1E) (Cremona et al., 2012; Psakhye and Jentsch, 2012). Following treatment with MMS, Smc5 sumoylation requires Mms21, and to a greater extent Nse5, Siz1 and Siz2 (Fig. 2C), and is consistent with the redundant nature of the three main E3 SUMO ligases commonly reported in *S. cerevisiae*. Components in both the cohesin and condensin complexes have similarly been found to require all three SUMO ligases for sumoylation (Almedawar et al., 2012; McAleenan et al., 2012; Takahashi et al., 2008). Currently it is not clear why all three SUMO ligases actively participate in damage-induced sumoylation of certain repair proteins, whereas other repair proteins, such as Rad52, have Siz2 as its dedicated E3 ligase. This may reflect ligase-specific recognition and binding to certain targets.

Compared with the other mutants, *nse5*-ts1 showed diminished binding to Nse6 suggesting that the Smc5/6 complex could become destabilized in this mutant background (Fig. 3E). Such destabilization might contribute to the loss of Smc5 sumoylation by impeding interactions between the complex and Siz1 or Siz2, which themselves are critical for Smc5 sumoylation. However, in order to conclusively address whether such interactions are direct in nature, *in vitro* studies with purified components of the Smc5/6 complex and the SUMO pathway will need to be performed. A speculative explanation could be that the Smc5/6 complex must be chromatin-bound in order for Smc5 sumoylation to occur, and that slight perturbations in complex stability leads to reduced chromatin binding. This theory is suggested based on the findings that targeting Siz2 to chromatin is sufficient to trigger the sumoylation of various HR proteins (Psakhye and Jentsch, 2012), and recent findings that Siz2 localization to sites DNA damage precedes RPA sumoylation (Chung et al., 2015).

In *nse5*-ts1 mutants, only minimal affects in the sumoylation of other Mms21 targets were detected. For example, the level of Yku70 and Smc2 sumoylation was reduced in cells carrying the *nse5*-ts1 allele, but levels were not lower than that observed in *mms21*-11 mutant cells (Fig. S4A,B). Our data support a hypothesis where the loss of viability in *nse5*-ts1 and *mms21*-11 mutants after MMS exposure is unrelated to Smc5 sumoylation and likely attributed to distinct properties arising in these mutants (Fig. 1D). The *nse5*-ts1 allele results in Smc5/6 complex destabilization as its interactions with Nse6 are disrupted (Fig. 3C,E) (Bustard et al., 2012). Such disruptions could impact ‘overall’ Smc5/6 functionality and lead to reduced fitness in response to DNA damage independently of Smc5 sumoylation. The combined loss of Smc5/6 integrity, together with the *mms21*-11 allele, which exhibits defects in the sumoylation targets involved in repair, such as Yku70, results in even a further loss of viability after exposure to DNA damage (Fig. 1A,D). We note however, even though this work suggests that Smc5 sumoylation offers no survival advantage after MMS-induced DNA damage, we cannot exclude the possibility that Smc5 sumoylation shares redundancy with a compensatory factor or pathway. As such, the impact of losing Smc5 sumoylation would only be revealed through additional mutagenesis. Moreover, our results do not exclude a role for Smc5 sumoylation or Nse5-SUMO interactions in response to other types of damage (Horigome et al., 2015).

By two-hybrid analysis, Nse5 interacts with Smt3/SUMO, however Nse5, itself, is not a target of sumoylation (Bustard et al., 2012), and no canonical SUMO interacting motifs (SIMs) are predicted in Nse5. SIMs are a short hydrophobic motif: (V/I)-x-(V/I)-(V/I) which may or may not be flanked by a stretch of acidic residues and located in unstructured, exposed areas of the protein where they insert into a hydrophobic groove on SUMO, facilitating hydrophobic interactions (Kerscher, 2007). However, it seems conceivable that Nse5 could contain non-canonical SUMO-interacting motifs, which have not yet been identified, that stabilize the non-covalent SUMO-Nse5 interaction. To date, there have been at least 20 ubiquitin-binding domains identified (Dikic et al., 2009), yet only one SUMO-interacting motif (SIM) has been described (Kerscher, 2007). It seems plausible that additional SUMO-interacting domains await identification.

Lastly, in human cells promyelocytic leukemia (PML) bodies form as a result of PML sumoylation and its colocalization with Smc5/6 (Brouwer et al., 2009; Potts and Yu, 2007). PML bodies function as ‘sumoylation centers’, however, *S.* *cerevisiae* lack PML bodies. Given the extensive interactions between the SUMO pathway components and Nse5 and Mms21, perhaps the Smc5/6 complex in budding yeast serves as a center for sumoylation in the absence of PML protein. We speculate that chromatin bound Smc5/6 facilitates the assembly of sumoylation enzymes at distinct loci to coordinate the sumoylation of target substrates.

## MATERIALS AND METHODS

### Yeast strains, plasmids, and antibodies

All experiments were performed at 25°C unless indicated otherwise. All strains used in these studies are listed in Table S1. Temperature sensitive strains *nse1*-5, *nse3*-1, *nse4*-2, *nse5*-ts1, *smc5*-6, and *smc6*-9 were derived from the Hieter lab (Ben-Aroya et al., 2008), and *mms21*-11 was received from the Zhao lab (Zhao and Blobel, 2005). The *smt3*-3kr (JC2996) and *smt3*-331 (JC2758) were derived from alleles provided by the Raught lab (Srikumar et al., 2013), and *His-FLAG-Smt3* (JC709) was received from the Johnson Lab (Johnson et al., 1997). All strains were backcrossed three times into the W303 *RAD5*+ background.

To integrate the Nse5 point mutants, the *NSE5* gene, its promoter and 3′UTR were sub-cloned into the integration vectors pRS304 and pRS306 giving J-053 and J-052 respectively Table S2 (Sikorski and Hieter, 1989). Mutagenesis using primers listed in Table S3 was performed on J-052 to generate the various mutants of *NSE5*, nse5* (Table S2). These vectors were subsequently integrated into the *URA3* locus by plasmid digestion with *Apa*I (Invitrogen) and transformation into wild-type W303 *RAD5*+ MATa (JC470). Next, in a separate strain, JC471, which is W303 *RAD5*+ MATα, the J-053 linearized vector was integrated at the *trp1-1* locus after *Xba*I digestion, which created *trp1*-1::*NSE5*::*TRP1.* This was followed by deletion of the endogenous *NSE5* via PCR-cassette based mutagenesis with a PCR product generated from primers containing the 5′ (FW) and 3′ (RV) UTR sequence of *NSE5* and *HIS3*-pFA6a. The resulting MATα strain, containing *trp1*-1::*NSE5*::*TRP1* and *nse5*Δ*::HIS3*, was then crossed to the various MATa strains containing the point mutants, *ura3*-1:nse5*:*URA3,* and NSE5+. Upon sporulation and tetrad dissection, haploid cells with the proper marker segregation, with growth on SC – Ura – His, but not SC – Ura – Trp, were verified by DNA sequencing at the University of Calgary sequencing facility to have NSE5, wild type or mutants, at the *URA3* locus but not at the endogenous or *TRP1* loci.

### Viability and drop assays

Drop assays were performed by growing cells overnight, then counting and adjusting cell density to the same concentration (roughly 1×10^7^ cells). Cells were diluted in 10-fold serial dilutions (unless noted), and 4 μl of each dilution was plated on YPAD plates with and without the indicated amounts of methyl methane sulfonate (MMS). Plates containing DNA damaging agents were always poured within 24 h of plating. Plates were incubated at 25°C (unless otherwise noted) for 4-5 days before photographing.

Cells were grown overnight at 25°C, and adjusted to 5×10^6^ cells/ml in the morning. Survival of transient MMS treatment was determined by treating cells with MMS (at the concentrations indicated) for one hour. 1000 cells were plated on YPAD, and percent survival was calculated by normalizing to untreated cells (plated prior to MMS treatment). Percent survival was calculated by normalizing to untreated cell growth.

### Yeast 2-hybrid (Y2H) analysis

Plasmids used for Y2H analysis are listed in Table S2. The *NSE5* genes were cloned into a pEG202-derived bait plasmid, creating Nse5-LexA fusion proteins, wild-type and mutant, which were under control of a galactose-inducible promoter (Aushubel et al., 1994). *NSE6*, *SMT3*, *UBC9*, *SIZ1*, *SIZ2*, *MMS21* and *CST9* were cloned into pJG4-6-derived prey vectors, creating a B42-activating domain fusion protein under the control of a galactose-inducible promoter. The Ubc9 mutants C93A and K153R were created by performing site-directed mutagenesis on plasmid J042. All constructs were confirmed by sequencing, and expression was confirmed by western blot analysis using anti-LexA (2-12, sc7544, Lot# K2006) and anti-HA (F-7, sc7392, Lot# H1914) antibodies from Santa Cruz Biotechnology at 1:2000 dilution.

Plasmids were transformed into JC1280 (Golemis et al., 1996). For drop assays, cells were grown up and plated in fivefold serial dilutions on plates containing 2% galactose and lacking histidine and tryptophan (to select for plasmids), and additionally leucine (to measure expression from *lexAop6-LEU2*). Plates were incubated for 5-6 days at 30°C before being photographed. Liquid culture assays were also performed in wild type (JC470) or *smt3*-331 (JC2758), where the reporter plasmid pSH18034 was also transformed (Aushubel et al., 1994). Protein-protein interactions were detected by quantitative β-galactosidase activity for permeabilized cells and represent the average of three independent experiments (Bjergbaek et al., 2005).

### Detection of sumoylated proteins

Myc-tagged Smc5 was used to detect Smc5 sumoylation were whole cell extracts were prepared by lysing cells with glass beads in lysis buffer (50 mM HEPES, 140 mM NaCl, 1 mM EDTA, and 1% Triton X-100). Extracts were incubated with anti-Myc coupled Dynabeads for 2 h at 4°C. Beads were washed by shaking at 14,000 rpm 1× in lysis buffer and 2× in wash buffer (100 mM Tris pH 8, 0.5% NP-40, 250 mM NaCl, 1 mM EDTA and 1.25 mM sodium deoxycholate) for 5 min each. Proteins were eluted from beads with SDS loading buffer and run on an 8% SDS-PAGE gel followed by western blotting with anti-Smt3 antibody (at 1:3000 dilution) a gift from Dr Xiaolan, Zhao Lab, Memorial Sloan Kettering. The blots were then stripped and reprobed with anti-Myc (9E10) antibody (at 1:1000 dilution) to serve as a loading control.

Due to limited anti-Smt3, an alternate method was used involving nickel-nitrilotriacetic acid (Ni-NTA) purification of His_8_-Smt3 as described in Wohlschlegel et al. (2004) with the following changes. Pellets of 2×10^9^ cells were washed with N-ethylmaleimide (Sigma) to preserve sumoylation. Proteins were incubated with Ni-NTA (Qiagen, Prod.# 1018244; Lot# 145022017) overnight at 4°C, followed by washing three times with 6 M guanidine hydrochloride, 100 mM NaH_2_PO_4_ (pH 8) and 0.05% Tween 20, and five times with 8 M urea, 100 mM NaH_2_PO_4_ (pH 6.3), and 0.05% Tween 20. Beads were resuspended in SDS loading buffer and run on 4-20% gradient gels (BioRad), or on low-bis gels (Bustard et al., 2012). Western blot analysis was perform with anti-Myc (9E10) at 1:1000 dilution

## References

[BIO018440C1] Albuquerque C. P., Wang G., Lee N. S., Kolodner R. D., Putnam C. D. and Zhou H. (2013). Distinct SUMO ligases cooperate with Esc2 and Slx5 to suppress duplication-mediated genome rearrangements. *PLoS Genet.* 9, e1003670 10.1371/journal.pgen.1003670 23935535 PMC3731205

[BIO018440C2] Almedawar S., Colomina N., Bermudez-Lopez M., Pocino-Merino I. and Torres-Rosell J. (2012). A SUMO-dependent step during establishment of sister chromatid cohesion. *Curr. Biol.* 22, 1576-1581. 10.1016/j.cub.2012.06.046 22771040

[BIO018440C3] Aushubel F. M., Brent R., Kinston R., Moore D., Seidman J. J., Smith J. and Struhl K. (1994). *Current Protocols in Molecular Biology*. New York: John Wiley and Sons.

[BIO018440C4] Ben-Aroya S., Coombes C., Kwok T., O'Donnell K. A., Boeke J. D. and Hieter P. (2008). Toward a comprehensive temperature-sensitive mutant repository of the essential genes of Saccharomyces cerevisiae. *Mol. Cell* 30, 248-258. 10.1016/j.molcel.2008.02.021 18439903 PMC4130347

[BIO018440C5] Bencsath K. P., Podgorski M. S., Pagala V. R., Slaughter C. A. and Schulman B. A. (2002). Identification of a multifunctional binding site on Ubc9p required for Smt3p conjugation. *J. Biol. Chem.* 277, 47938-47945. 10.1074/jbc.M207442200 12354763

[BIO018440C6] Bermudez-Lopez M., Pocino-Merino I., Sanchez H., Bueno A., Guasch C., Almedawar S., Bru-Virgili S., Gari E., Wyman C., Reverter D. et al. (2015). ATPase-dependent control of the Mms21 SUMO ligase during DNA repair. *PLoS Biol.* 13, e1002089 10.1371/journal.pbio.1002089 25764370 PMC4357442

[BIO018440C7] Bernier-Villamor V., Sampson D. A., Matunis M. J. and Lima C. D. (2002). Structural basis for E2-mediated SUMO conjugation revealed by a complex between ubiquitin-conjugating enzyme Ubc9 and RanGAP1. *Cell* 108, 345-356. 10.1016/S0092-8674(02)00630-X 11853669

[BIO018440C8] Biggins S., Bhalla N., Chang A., Smith D. L. and Murray A. W. (2001). Genes involved in sister chromatid separation and segregation in the budding yeast Saccharomyces cerevisiae. *Genetics* 159, 453-470. 11606525 10.1093/genetics/159.2.453 PMC1461834

[BIO018440C9] Bjergbaek L., Cobb J. A., Tsai-Pflugfelder M. and Gasser S. M. (2005). Mechanistically distinct roles for Sgs1p in checkpoint activation and replication fork maintenance. *EMBO J.* 24, 405-417. 10.1038/sj.emboj.7600511 15616582 PMC545806

[BIO018440C10] Brouwer A. K., Schimmel J., Wiegant J. C. A. G., Vertegaal A. C. O., Tanke H. J. and Dirks R. W. (2009). Telomeric DNA mediates de novo PML body formation. *Mol. Biol. Cell* 20, 4804-4815. 10.1091/mbc.E09-04-0309 19793919 PMC2777109

[BIO018440C11] Bustard D. E., Menolfi D., Jeppsson K., Ball L. G., Dewey S. C., Shirahige K., Sjogren C., Branzei D. and Cobb J. A. (2012). During replication stress, non-SMC element 5 (NSE5) is required for Smc5/6 protein complex functionality at stalled forks. *J. Biol. Chem.* 287, 11374-11383. 10.1074/jbc.M111.336263 22303010 PMC3322872

[BIO018440C12] Cheng C.-H., Lo Y.-H., Liang S.-S., Ti S.-C., Lin F.-M., Yeh C.-H., Huang H.-Y. and Wang T.-F. (2006). SUMO modifications control assembly of synaptonemal complex and polycomplex in meiosis of Saccharomyces cerevisiae. *Genes Dev.* 20, 2067-2081. 10.1101/gad.1430406 16847351 PMC1536058

[BIO018440C13] Chung D. K. C., Chan J. N. Y., Strecker J., Zhang W., Ebrahimi-Ardebili S., Lu T., Abraham K. J., Durocher D. and Mekhail K. (2015). Perinuclear tethers license telomeric DSBs for a broad kinesin- and NPC-dependent DNA repair process. *Nat. Commun.* 6, 7742 10.1038/ncomms8742 26205667

[BIO018440C14] Cremona C. A., Sarangi P., Yang Y., Hang L. E., Rahman S. and Zhao X. (2012). Extensive DNA damage-induced sumoylation contributes to replication and repair and acts in addition to the mec1 checkpoint. *Mol. Cell* 45, 422-432. 10.1016/j.molcel.2011.11.028 22285753 PMC3340930

[BIO018440C15] Dikic I., Wakatsuki S. and Walters K. J. (2009). Ubiquitin-binding domains - from structures to functions. *Nat. Rev. Mol. Cell Biol.* 10, 659-671. 10.1038/nrm2767 19773779 PMC7359374

[BIO018440C16] Duan X., Sarangi P., Liu X., Rangi G. K., Zhao X. and Ye H. (2009a). Structural and functional insights into the roles of the Mms21 subunit of the Smc5/6 complex. *Mol. Cell* 35, 657-668. 10.1016/j.molcel.2009.06.032 19748359 PMC2993495

[BIO018440C17] Duan X., Yang Y., Chen Y.-H., Arenz J., Rangi G. K., Zhao X. and Ye H. (2009b). Architecture of the Smc5/6 Complex of Saccharomyces cerevisiae Reveals a Unique Interaction between the Nse5-6 Subcomplex and the Hinge Regions of Smc5 and Smc6. *J. Biol. Chem.* 284, 8507-8515. 10.1074/jbc.M809139200 19141609 PMC2659209

[BIO018440C18] Ferreira H. C., Luke B., Schober H., Kalck V., Lingner J. and Gasser S. M. (2011). The PIAS homologue Siz2 regulates perinuclear telomere position and telomerase activity in budding yeast. *Nat. Cell Biol.* 13, 867-874. 10.1038/ncb2263 21666682

[BIO018440C19] Gareau J. R. and Lima C. D. (2010). The SUMO pathway: emerging mechanisms that shape specificity, conjugation and recognition. *Nat. Rev. Mol. Cell Biol.* 11, 861-871. 10.1038/nrm3011 21102611 PMC3079294

[BIO018440C20] Geiss-Friedlander R. and Melchior F. (2007). Concepts in sumoylation: a decade on. *Nat. Rev. Mol. Cell Biol.* 8, 947-956. 10.1038/nrm2293 18000527

[BIO018440C21] Golemis E. A., Gyuris J. and Brent R. (1996). Interaction Trap/two hybrid system to identify interacting proteins. In *Current Protocals in Molecular Biology* (ed. Ausubel F. M., Brent R., Kingston R. E., Moore D. D., Seidman J. G., Smith J. A. and Struhl K.), pp. 20.21.21-20.21.23. New York:John Wiley & Sons, Inc.

[BIO018440C22] Hazbun T. R., Malmstrom L., Anderson S., Graczyk B. J., Fox B., Riffle M., Sundin B. A., Aranda J. D., McDonald W. H., Chiu C.-H. et al. (2003). Assigning function to yeast proteins by integration of technologies. *Mol. Cell* 12, 1353-1365. 10.1016/S1097-2765(03)00476-3 14690591

[BIO018440C123] Horigome C., Bustard D., Marcomini I., Delgoshaie N., Tsai-Pflugfelder M., Cobb J. A. and Gasser S. M. (2015). PolySUMOylation by Siz2 and Mms21 triggers relocation of DNA breaks to nuclear pores through the Slx5/Slx8 STUbL. *Genes Dev.* 30, 931-945. 10.1101/gad.277665.116 27056668 PMC4840299

[BIO018440C23] Jeppsson K., Kanno T., Shirahige K. and Sjogren C. (2014). The maintenance of chromosome structure: positioning and functioning of SMC complexes. *Nat. Rev. Mol. Cell Biol.* 15, 601-614. 10.1038/nrm3857 25145851

[BIO018440C24] Johnson E. S. and Blobel G. (1997). Ubc9p is the conjugating enzyme for the ubiquitin-like protein Smt3p. *J. Biol. Chem.* 272, 26799-26802. 10.1074/jbc.272.43.26799 9341106

[BIO018440C25] Johnson E. S. and Gupta A. A. (2001). An E3-like factor that promotes SUMO conjugation to the yeast septins. *Cell* 106, 735-744. 10.1016/S0092-8674(01)00491-3 11572779

[BIO018440C26] Johnson E. S., Schwienhorst I., Dohmen R. J. and Blobel G. (1997). The ubiquitin-like protein Smt3p is activated for conjugation to other proteins by an Aos1p/Uba2p heterodimer. *EMBO J.* 16, 5509-5519. 10.1093/emboj/16.18.5509 9312010 PMC1170183

[BIO018440C27] Kerscher O. (2007). SUMO junction-what's your function? New insights through SUMO-interacting motifs. *EMBO Rep.* 8, 550-555. 10.1038/sj.embor.7400980 17545995 PMC2002525

[BIO018440C28] Klug H., Xaver M., Chaugule V. K., Koidl S., Mittler G., Klein F. and Pichler A. (2013). Ubc9 sumoylation controls SUMO chain formation and meiotic synapsis in Saccharomyces cerevisiae. *Mol. Cell* 50, 625-636. 10.1016/j.molcel.2013.03.027 23644018

[BIO018440C29] Mahajan R., Gerace L. and Melchior F. (1998). Molecular characterization of the SUMO-1 modification of RanGAP1 and its role in nuclear envelope association. *J. Cell Biol.* 140, 259-270. 10.1083/jcb.140.2.259 9442102 PMC2132567

[BIO018440C30] Matunis M. J., Wu J. and Blobel G. (1998). SUMO-1 modification and its role in targeting the Ran GTPase-activating protein, RanGAP1, to the nuclear pore complex. *J. Cell Biol.* 140, 499-509. 10.1083/jcb.140.3.499 9456312 PMC2140169

[BIO018440C31] McAleenan A., Cordon-Preciado V., Clemente-Blanco A., Liu I.-C., Sen N., Leonard J., Jarmuz A. and Aragon L. (2012). SUMOylation of the alpha-kleisin subunit of cohesin is required for DNA damage-induced cohesion. *Curr. Biol.* 22, 1564-1575. 10.1016/j.cub.2012.06.045 22771042

[BIO018440C32] Palecek J., Vidot S., Feng M., Doherty A. J. and Lehmann A. R. (2006). The Smc5-Smc6 DNA repair complex: bridging of the Smc5-Smc6 heads by the KLEISIN, Nse4, and non-Kleisin subunits. *J. Biol. Chem.* 281, 36952-36959. 10.1074/jbc.M608004200 17005570

[BIO018440C33] Potts P. R. and Yu H. (2007). The SMC5/6 complex maintains telomere length in ALT cancer cells through SUMOylation of telomere-binding proteins. *Nat. Struct. Mol. Biol.* 14, 581-590. 10.1038/nsmb1259 17589526

[BIO018440C34] Psakhye I. and Jentsch S. (2012). Protein group modification and synergy in the SUMO pathway as exemplified in DNA repair. *Cell* 151, 807-820. 10.1016/j.cell.2012.10.021 23122649

[BIO018440C35] Schwarz S. E., Matuschewski K., Liakopoulos D., Scheffner M. and Jentsch S. (1998). The ubiquitin-like proteins SMT3 and SUMO-1 are conjugated by the UBC9 E2 enzyme. *Proc. Natl. Acad. Sci. USA* 95, 560-564. 10.1073/pnas.95.2.560 9435231 PMC18459

[BIO018440C36] Sikorski R. S. and Hieter P. (1989). A system of shuttle vectors and yeast host strains designed for efficient manipulation of DNA in Saccharomyces cerevisiae. *Genetics* 122, 19-27. 2659436 10.1093/genetics/122.1.19 PMC1203683

[BIO018440C37] Srikumar T., Lewicki M. C., Costanzo M., Tkach J. M., van Bakel H., Tsui K., Johnson E. S., Brown G. W., Andrews B. J., Boone C. et al. (2013). Global analysis of SUMO chain function reveals multiple roles in chromatin regulation. *J. Cell Biol.* 201, 145-163. 10.1083/jcb.201210019 23547032 PMC3613684

[BIO018440C38] Takahashi Y., Dulev S., Liu X., Hiller N. J., Zhao X. and Strunnikov A. (2008). Cooperation of sumoylated chromosomal proteins in rDNA maintenance. *PLoS Genet.* 4, e1000215 10.1371/journal.pgen.1000215 18846224 PMC2563031

[BIO018440C39] Tapia-Alveal C., Lin S.-J. and O'Connell M. J. (2014). Functional interplay between cohesin and Smc5/6 complexes. *Chromosoma* 123, 437-445. 10.1007/s00412-014-0474-9 24981336 PMC4169997

[BIO018440C40] Wohlschlegel J. A., Johnson E. S., Reed S. I. and Yates J. R. III (2004). Global analysis of protein sumoylation in Saccharomyces cerevisiae. *J. Biol. Chem.* 279, 45662-45668. 10.1074/jbc.M409203200 15326169

[BIO018440C41] Zhao X. and Blobel G. (2005). A SUMO ligase is part of a nuclear multiprotein complex that affects DNA repair and chromosomal organization. *Proc. Natl. Acad. Sci. USA* 102, 4777-4782. 10.1073/pnas.0500537102 15738391 PMC555716

